# Association between sleep duration, sleep trouble and all-cause mortality in individuals with hyperuricemia in the United States

**DOI:** 10.3389/fpubh.2025.1521372

**Published:** 2025-03-26

**Authors:** Yuanhui Dai, Xiangyu Sun, Ge Zhang, Chunying Cui, Xiaoli Wu, Yierzhati Aizezi, Kaisaierjiang Kadier

**Affiliations:** ^1^Department of Cardiology, First Affiliated Hospital of Xinjiang Medical University, Urumqi, China; ^2^Clinical Medicine College, , Xinjiang Medical University, Urumqi, China; ^3^Department of Cardiology, First Affiliated Hospital of Zhengzhou University, Zhengzhou, China; ^4^Department of Emergency Medicine, Jining First People's Hospital, Shandong First Medical University, Jining, China; ^5^Critical Medicine Center, The First Affiliated Hospital of Xinjiang Medical University, Urumqi, China

**Keywords:** hyperuricemia, sleep duration, sleep trouble, all-cause mortality, prognosis, NHANES

## Abstract

**Objectives:**

Despite the crucial role of sleep quality in hyperuricemia onset and progression, there is limited evidence on sleep interventions to improve outcomes for hyperuricemic individuals. This study aims to investigate the effects of sleep duration and sleep difficulties on all-cause mortality in this population.

**Materials and methods:**

We conducted a secondary analysis of the National Health and Nutrition Examination Survey (NHANES) data from 2007 to 2018, including 5,837 participants. We employed weighted multivariable Cox proportional hazard models to evaluate the independent predictive value of sleep duration and trouble for all-cause mortality. Restricted cubic splines and segmented Cox proportional hazard models were used to examine threshold effects.

**Results:**

During a mean follow-up of 6.5 years, 906 participants experienced all-cause mortality. After adjusting for confounders, both short (< 7 h; HR = 1.25; 95%CI: 1.04, 1.51; *p* = 0.018) and long (>9 h; HR = 1.50; 95%CI: 1.10, 2.04; *p* = 0.011) sleep durations were associated with increased all-cause mortality. The threshold analysis identified an optimal sleep duration of 7.23 h, and when sleep duration was below 7.23 h, it was inversely related to mortality (HR: 0.879; 95% CI: 0.788, 0.981; *p* = 0.022). Conversely, when sleep duration exceeded 7.23 h, it was positively associated with mortality (HR: 1.187; 95% CI: 1.066, 1.320; *p* = 0.002).

**Conclusion:**

Sleep duration is U-shapedly associated with all-cause mortality among individuals with hyperuricemia in the United States. However sleep trouble was not associated with all-cause mortality. Maintaining optimal sleep duration helps improve the prognostic survival rates of those with hyperuricemia.

## 1 Introduction

Uric acid (UA) is primarily produced by the liver and represents the final product of purine catabolism. When UA is synthesized too much or excreted too little through the kidney and intestine, hyperuricemia may result (1). A broad survey conducted in the U.S. between 2015 and 2016 revealed that 20.1% of adults were affected by hyperuricemia, with a prevalence of 27.2% among those aged 65 and older (2). Studies have demonstrated that hyperuricemia is linked to the development of diseases across various organs and systems in the body. This includes cardiovascular disease (CVD) in the circulatory system, kidney diseases in the urinary system, diabetes mellitus (DM) in the endocrine system, as well as gout in the musculoskeletal system (3, 4). Furthermore, serum UA levels measured upon hospital admission are a strong independent predictor of negative cardiovascular outcomes (5). Importantly, hyperuricemia is not only linked to a higher frequency of related diseases but also has a significant independent association with both cardiovascular and all-cause mortality rates (6).

Research indicates that the sleep health plays a significant role in both the onset and progression of hyperuricemia (7–10). It involves getting enough sleep and a good quality of sleep. Numerous studies have explored the link between sleep and hyperuricemia. According to data from the China Health and Nutrition Survey (CHNS), people who sleep less are at an increased risk for hyperuricemia (11). Additionally, another cross-sectional study from the Chinese population found that individuals with longer sleep durations tend to have a reduced incidence of hyperuricemia when compared to those with shorter sleep durations (12). In the Mediterranean older population, sleep duration was negatively correlated with serum UA levels (13), while in the Korean female population, the association became U-shaped (14), and this difference may be due to race, age, and sex. At present, some known molecular mechanisms also support these findings, when sleep deprivation (15), purine and other protein breakdown products will increase, resulting in increased synthesis of UA, and when lack of sleep, it will increase the body’s insulin resistance (7), which further promotes the occurrence and development of hyperuricemia (8). In addition, insufficient or excessive sleep has been shown to be associated with conditions such as hyperlipidemia, hypertension, DM, CVD, and obesity (16, 17), which increase the risk of all-cause mortality in individuals to some extent. This is also confirmed in the study concerning sleep duration and all-cause mortality, that is, the relationship between the two is U-shaped, and insufficient or too long sleep will increase all-cause mortality (18). Although existing research has investigated the link between hyperuricemia, sleep, and all-cause mortality, the effect of sleep duration and sleep trouble on all-cause mortality in hyperuricemic individuals has not been clarified. Therefore, this study delves into the effect of sleep duration and sleep trouble regarding all-cause mortality in those with hyperuricemia, and gives recommendations for optimal sleep duration for hyperuricemic individuals through threshold effect analysis. This will help to clarify the health benefits of intervening sleep on hyperuricemic individuals, provide a scientific basis for clinical intervention, and improve the health outcomes of hyperuricemic individuals.

## 2 Materials and methods

### 2.1 Study design and population

**Figure 1:**
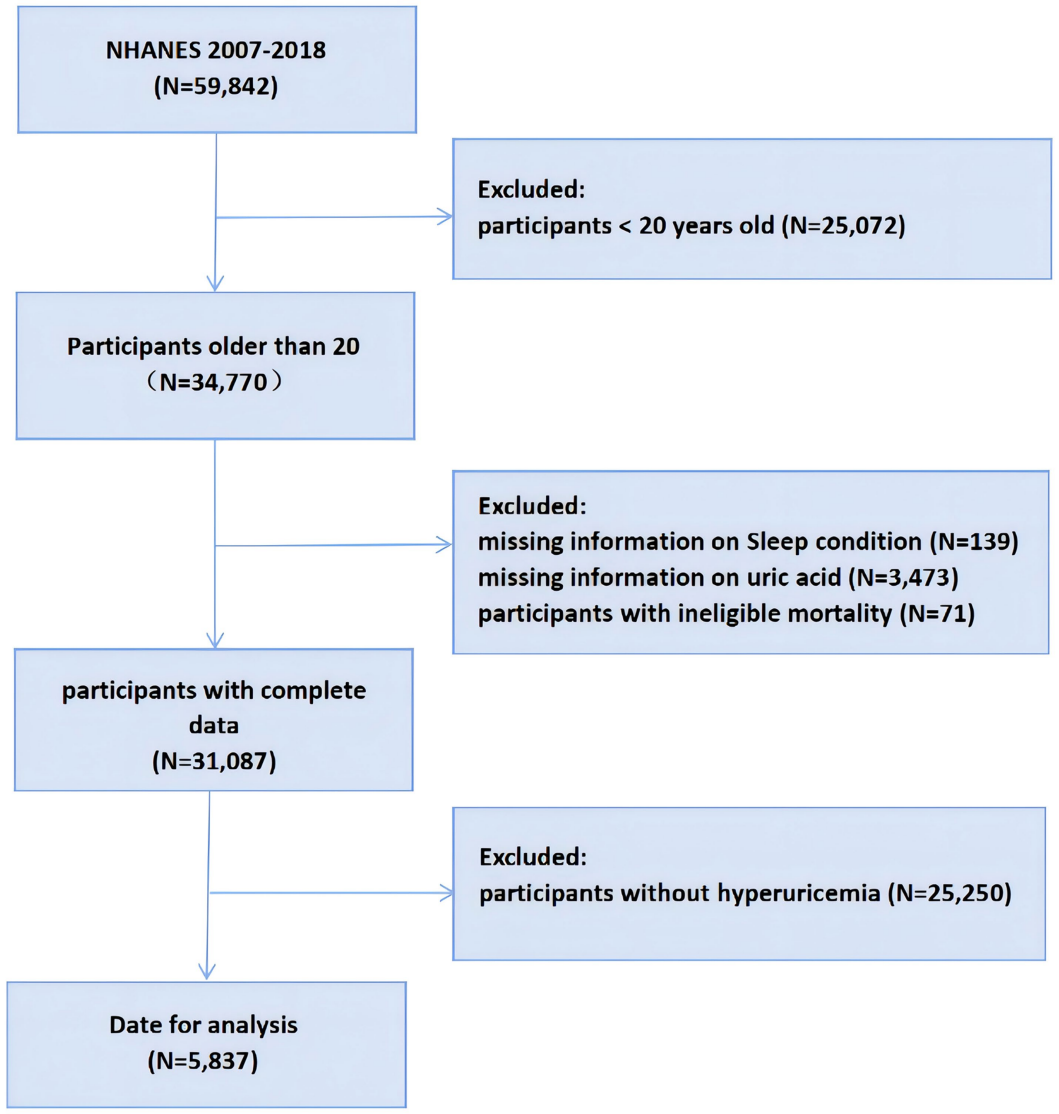
Flowchart of the participants selection from NHANES 2007–2018. NHANES, National Health and Nutrition Examination Survey.

This study analyzed data from the National Health and Nutrition Examination Survey (NHANES) spanning six cycles from 2007 to 2018. NHANES, established in the 1960s, aims to provide a comprehensive understanding of the U.S. population by systematically collecting data on their health and nutrition. Participants are randomly chosen from a range of populations across the United States to provide a representative sample. The basic content of NHANES includes demographic information, health status, nutrition data, and physical examination data. NHANES data are widely utilized for public health research, health guidance, and early disease prevention. From 2007 to 2018, the NHANES included 59,842 participants, where we removed individuals under the age of 20 (*N* = 25,072), in addition to deaths with ineligible data (*N* = 71), missing data on UA (*N* = 3,473), missing sleep duration and sleep trouble (*N* = 139), and participants who did not suffer from hyperuricemia (*N* = 25,250) in participants. A total of 5,837 participants were ultimately enrolled in the study, as shown in Figure 1.

### 2.2 Measurement of serum UA and definition of hyperuricemia

Serum samples from participants were stored at low temperatures until transported to a collaborating laboratory for analysis. Serum UA was measured by colorimetry using the Beckman Coulter UniCel® DxC800 Synchronizer from 2007 to 2016 and the Roche Cobas 6,000 chemistry analyzer from 2017 to 2018 (19). Hyperuricemia is identified when serum UA levels surpass 7.0 mg/dL in males and 6.0 mg/dL in females (20).

### 2.3 Sleep duration and sleep trouble

Both sleep duration and sleep trouble were recorded by self-reporting. Sleep hours were recorded from 2007 to 2016 through the question “How much sleep do you usually get at night on weekdays or workdays?” From 2017 to 2018 through the questions “Number of hours usually sleep on weekdays or workdays.” and “Number of hours usually sleep on weekends or non workdays.” Sleep duration was recorded. Three categories were used to categorize the amount of sleep: normal (between 7 and 9 h), short (less than 7 h), and long (more than 9 h) (21). Assessing whether the participants have trouble sleeping by asking the question, “Have you ever told a doctor or other health professional that you have trouble sleeping.” More detailed details of the issue can be found in Supplementary Tables 1, 2.

### 2.4 Determination of mortality outcomes

To ascertain the all-cause mortality of participants, we used National Death Index (NDI) records prior to December 31, 2019, which correlated participants’ death certificate records with public mortality files provided by NCHS through a probability matching algorithm.

### 2.5 Covariates

Previous studies on hyperuricemia and sleep provided a basis for the covariates chosen for this investigation. Demographic characteristics were obtained through standardized household interviews, these include age, gender (male or female), race (Non-Hispanic Black, Non-Hispanic White, Mexican American, and others), education (less than high school, high school and above high school), insurance coverage (yes or no), and poverty-income ratio (PIR; < 1.3, 1.3–3.5 and > 3.5). Part of the participants’ health status was obtained through self-report questionnaires, these include smoking status (never, now and former), alcohol consumption (never, former, mild–moderate and heavy), cancer (yes or no), gout (yes or no), and CVD (yes or no). The measurement of UA levels has been described in detail above. Physical activity was assessed using the Physical Activity Questionnaire (PAQ). This encompasses four categories of physical activity, with each category scored based on NHANES guidelines for the metabolic equivalent of task (MET) (22). Based on the U.S. Physical Activity Guidelines, participants were classified into four levels of physical activity: Extremely highly active, highly active, Low-active, and inactive. The specific criteria for these classifications are detailed in Supplementary Table 3. Body Mass Index (BMI) was measured using a standardized scheme in the mobile examination center. The definitions of hypertension, DM, and chronic kidney disease (CKD) refer to similar previous studies, and the detailed diagnostic criteria are shown in Supplementary Table 4 (23, 24).

### 2.6 Statistical analyses

All statistical analyses of this study were followed “The Strengthening the Reporting of Observational Studies in Epidemiology (STROBE) statement: guidelines for reporting observational studies.” Because NHANES uses a complex multi-stage stratified probability survey design, this study integrates survey design variables and sampling weights to avoid analytical bias and thus provide accurate estimates. Continuous variables were represented by mean and standard deviation (SD) to illustrate their central tendency and variability, whereas categorical variables were depicted through weighted percentages (95% confidence intervals, 95% CI) to reflect their distribution across different groups. One-way analysis of variance (ANOVA) was employed for continuous variables to evaluate variations among groups, while categorical variables were analyzed using the Rao-Scott chi-square test to accurately evaluate relationships between categorical variables. To assess the independent predictive value of sleep duration and sleep trouble for all-cause mortality, we developed a multivariable Cox proportional hazard model. Model 1 adjusting for age, sex and race. Model 2 further adjusted for alcohol consumption, Smoking status, education, PIR, insurance coverage, BMI, physical activity level and UA. Model 3 further adjusts for cancer, gout, DM, hypertension, CVD and CKD on the basis of Model 2.

In order to investigate the dose–response relationship between sleep duration and all-cause mortality, we employed the Cox proportional hazard model fitted by restricted cubic splines (RCS). If RCS indicates a non-linear association between the two, then, a “segmented” package based on likelihood ratio test and bootstrap resampling method is further used to determine the inflection point (25). Specifically, we set three knots at the 10th, 50th, and 90th percentiles of the data, respectively, to capture the non - linear association between sleep duration and all - cause mortality. Furthermore, we employed a segmented Cox proportional - hazards regression model on both sides of the inflection point to explore the impact of sleep duration on either side of the inflection point on all - cause mortality. To explore differences among subgroups, we performed stratified analyses by age, sex, and race in a fully adjusted model, and evaluated interactions between variables by detecting likelihood ratios, with *p*-values less than 0.05 considered statistically significant.

Statistical analyses for this study were performed using R software (version 4.1.3), and missing values of covariables were processed by using missforest. Supplementary Table 5 shows the missing comprehensive data. Sensitivity analysis was conducted based on the following criteria: (1) only participants with complete covariate data were included; (2) participants with cancer were excluded.

## 3 Results

### 3.1 Baseline characteristics

This study analyzed 5,837 participants, with an average baseline age of 51.30 ± 0.34 years and a mean UA level of 7.50 ± 0.02 mg/dL. Of these, 44.30% were female (95% CI 41.22–47.38), and the average BMI was 32.69 ± 0.15 kg/m^2^. The all-cause mortality for the cohort was 11.36% (95% CI: 10.04–12.67). All-cause mortality was 10.33% (95% CI: 8.96–11.70) for those with normal sleep duration, 12.20% (95% CI: 10.20–14.21) for those with short sleep duration, and 17.53% (95% CI: 13.19–21.86) for those with prolonged sleep duration. The groups’ all-cause mortality varied significantly, with the group with normal sleep duration having the lowest all-cause mortality. Demographically, those with normal sleep duration had a higher likelihood of being older men, as well as those with moderate insurance coverage, higher education levels, and higher economic income than those with short or long sleep duration. In terms of health status, participants with normal sleep duration were more likely to have a moderate BMI, lower UA levels, lower prevalence of gout, lower prevalence of DM, lower prevalence of hypertension, lower smoking levels, and lower prevalence of CVD. Among all participants, 31.19% (95% CI: 28.74–33.65) reported sleep trouble. Among individuals with shorter sleep duration, 38.24% (95% CI: 35.33–41.14) reported sleep trouble, while among those with longer sleep duration, 35.75% (95% CI: 28.79–42.70) reported sleep trouble. Table 1 displays all of the participants’ basic characteristics.

**Table 1:** The variable order is currently disorganized, and I will re-upload a file with the correct variable order without modifying the data.

	Sleep time				
Characters	Overall	Normal (7–9 hours)	Short (< 7 hours)	Long (>9 hours)	*p*-value
	(*N* = 5,837)	(*N* = 3,362)	(*N* = 2,083)	(*N* = 392)	
Age-year	51.30±0.34	51.98±0.44	49.11±0.46	55.98±1.44	< 0.001
Age-year (%)					< 0.001
20-39	29.98 (27.51–32.46)	28.81 (26.44–31.19)	32.51 (29.72–35.29)	28.61 (22.08–35.14)	
40-59	33.59 (30.74–36.44)	32.50 (30.17–34.83)	38.55 (35.87–41.23)	18.15 (13.03–23.27)	
≧ 60	36.42 (33.80–39.05)	38.69 (36.32–41.06)	28.94 (26.51–31.38)	53.24 (45.87–60.62)	
Sex (%)					< 0.001
Female	44.30 (41.22–47.38)	44.30 (42.03–46.57)	41.62 (38.96–44.28)	58.79 (52.60–64.98)	
Male	55.70 (51.89–59.51)	55.70 (53.43–57.97)	58.38 (55.72–61.04)	41.21 (35.02–47.40)	
Race (%)					< 0.001
Non-Hispanic White	68.53 (62.07–74.98)	72.27 (69.24–75.31)	61.98 (57.83–66.12)	64.73 (58.52–70.94)	
Non-Hispanic Black	12.71 (11.10–14.32)	9.50 (7.94–11.06)	18.44 (15.44–21.44)	15.30 (10.98–19.62)	
Mexican American	6.50 (5.24–7.75)	6.86 (5.39–8.32)	5.77 (4.32–7.23)	6.65 (3.68–9.62)	
Others	12.27 (10.95–13.59)	11.37 (9.79–12.95)	13.81 (11.88–15.74)	13.32 (10.09–16.55)	
Education (%)					< 0.001
Less than high school	16.18 (14.60–17.75)	14.47 (12.91–16.04)	18.14 (16.35–19.93)	23.39 (18.26–28.53)	
High school	25.16 (22.72–27.59)	24.86 (22.80–26.91)	25.08 (22.61–27.55)	28.73 (22.45–35.01)	
Above high school	58.67 (54.63–62.70)	60.67 (58.04–63.30)	56.79 (54.02–59.55)	47.88 (41.15–54.61)	
Poverty-income ratio (%)					< 0.001
< 1.3	20.32 (18.82–21.82)	17.34 (15.74–18.94)	23.72 (21.38–26.05)	33.16 (26.67–39.65)	
1.3-3.5	42.06 (39.20–44.92)	43.29 (40.92–45.65)	39.26 (36.34–42.17)	44.36 (37.92–50.80)	
> 3.5	37.62 (33.83–41.41)	39.37 (36.59–42.15)	37.03 (33.30–40.75)	22.48 (14.02–30.94)	
Insurance (%)					0.044
No	15.20 (13.87–16.53)	14.57 (12.92–16.22)	17.03 (15.10–18.95)	11.92 (8.03–15.81)	
Yes	84.80 (79.31–90.28)	85.43 (83.78–87.08)	82.97 (81.05–84.90)	88.08 (84.19–91.97)	
Smoking status (%)					< 0.001
Never	52.40 (49.14–55.65)	52.47 (50.07–54.88)	52.25 (49.54–54.96)	52.39 (44.64–60.14)	
Now	17.33 (15.45–19.22)	15.17 (13.27–17.08)	21.10 (18.62–23.58)	19.55 (13.95–25.15)	
Former	30.27 (27.61–32.93)	32.35 (30.07–34.64)	26.65 (24.10–29.20)	28.06 (21.66–34.46)	
Alcohol consumption status (%)					0.003
Never	10.94 (9.59–12.29)	10.80 (9.30–12.30)	10.49 (8.83–12.15)	14.92 (11.17–18.67)	
Former	13.98 (12.34–15.61)	13.21 (11.55–14.86)	15.68 (13.94–17.43)	12.82 (9.18–16.45)	
Mild-Moderate	35.31 (32.30–38.32)	36.40 (33.58–39.22)	31.98 (28.88–35.08)	41.99 (34.41–49.56)	
Heavy	39.77 (36.77–42.77)	39.60 (36.95–42.24)	41.85 (39.34–44.36)	30.28 (23.13–37.43)	
PA (%)					< 0.001
Inactive	26.71 (24.68–28.74)	26.11 (24.08–28.14)	24.41 (21.72–27.10)	45.52 (37.78–53.26)	
Low-active	14.95 (13.31–16.58)	15.73 (14.03–17.43)	13.91 (12.01–15.81)	12.34 (9.30–15.37)	
Highly active	15.22 (13.54–16.90)	15.88 (14.13–17.63)	14.23 (12.17–16.29)	13.67 (7.66–19.67)	
Extremely highly active	43.12 (39.96–46.28)	42.27 (39.79–44.76)	47.45 (44.26–50.65)	28.48 (22.22–34.74)	
BMI (kg/m²)	32.69±0.15	32.50±0.18	33.21±0.24	31.88±0.54	0.017
Uric acid (mg/dL)	7.50±0.02	7.48±0.02	7.53±0.03	7.48±0.07	0.222
DM (%)					0.075
No	80.88 (75.62–86.14)	81.87 (80.13–83.61)	79.89 (77.59–82.18)	75.88 (69.35–82.41)	
Yes	19.12 (17.41–20.83)	18.13 (16.39–19.87)	20.11 (17.82–22.41)	24.12 (17.59–30.65)	
Hypertension (%)					0.313
No	30.55 (27.85–33.24)	31.36 (29.10–33.62)	29.45 (26.82–32.07)	27.98 (21.95–34.01)	
Yes	69.45 (65.01–73.90)	68.64 (66.38–70.90)	70.55 (67.93–73.18)	72.02 (65.99–78.05)	
Gout (%)					0.512
No	90.30 (84.77–95.83)	90.47 (89.16–91.78)	90.45 (88.86–92.04)	87.67 (81.46–93.89)	
Yes	9.70 (8.52–10.88)	9.53 (8.22–10.84)	9.55 (7.96–11.14)	12.33 (6.11–18.54)	
Cancer (%)					0.002
No	87.72 (82.40–93.04)	87.03 (85.65–88.41)	89.93 (88.14–91.72)	82.88 (78.57–87.19)	
Yes	12.28 (10.96–13.61)	12.97 (11.59–14.35)	10.07 (8.28–11.86)	17.12 (12.81–21.43)	
CVD (%)					< 0.001
No	85.72 (80.23–91.21)	86.49 (84.97–88.01)	86.19 (84.10–88.28)	75.11 (69.35–80.87)	
Yes	14.28 (12.86–15.70)	13.51 (11.99–15.03)	13.81 (11.72–15.90)	24.89 (19.13–30.65)	
CKD (%)					< 0.001
No	71.67 (66.99–76.35)	70.29 (68.31–72.28)	77.23 (75.15–79.32)	55.84 (49.13–62.55)	
Yes	28.33 (26.12–30.55)	29.71 (27.72–31.69)	22.77 (20.68–24.85)	44.16 (37.45–50.87)	
sleep trouble (%)					< 0.001
No	68.81 (64.39–73.22)	72.88 (70.66–75.11)	61.76 (58.86–64.67)	64.25 (57.30–71.21)	
Yes	31.19 (28.74–33.65)	27.12 (24.89–29.34)	38.24 (35.33–41.14)	35.75 (28.79–42.70)	
Mortstat (%)					0.004
alive	88.64 (83.26–94.03)	89.67 (88.30–91.04)	87.80 (85.79–89.80)	82.47 (78.14–86.81)	
deceased	11.36 (10.04–12.67)	10.33 (8.96–11.70)	12.20 (10.20–14.21)	17.53 (13.19–21.86)	

Abbreviations: BMI, Body Mass Index; DM, Diabetes mellitus; PA, Physical Activity; CKD, Chronic kidney disease; CVD, Cardiovascular disease. Continuous variables are presented as weighted mean ± standard deviation, while categorical variables are presented as weighted percentages (95% confidence interval). Continuous variables were compared by weighted one-way ANOVA, while categorical variables were compared by weighted Rao-Scott chi-square test.

### 3.2 Sleep duration, sleep trouble, and all-cause mortality in hyperuricemic individuals

Over a follow-up period of 6.5 years on average, a total of 906 participants died. As shown in Table 2, in models 1, 2, and fully adjusted models, participants with shorter and longer sleep durations had higher mortality risk ratios in contrast to those with normal sleep durations. In the fully adjusted model, compared to those with normal sleep duration, participants with shorter sleep duration had a mortality HR of 1.25 (95% CI: 1.04, 1.51; *p* = 0.018), while those with longer sleep duration had a mortality HR of 1.50 (95% CI: 1.10, 2.04; *p* = 0.011). In model 1, participants with sleep trouble have a higher mortality HR compared to those without sleep trouble, with a *p*-value of 0.008, indicating statistical significance. However, in models 2 and 3, after adjusting for confounding factors, this association loses its statistical significance.

**Table 2:** Cox proportional hazard model for sleep duration, sleep trouble and all-cause mortality in hyperuricemic individuals.

		Model 1		Model 2		Model 3	
	Case/participants	HR (95%CI)	*p* value	HR (95%CI)	*p* value	HR (95%CI)	*p* value
Sleep time							
Normal (7–9 h)	497/3,362	Reference		Reference		Reference	
Short (< 7 h)	324/2,083	1.34 (1.10–1.65)	0.004	1.24 (1.02–1.50)	0.028	1.25 (1.04–1.51)	0.018
Long (>9 h)	85/392	1.97 (1.51–2.57)	<0.0001	1.47 (1.05–2.07)	0.026	1.50 (1.10–2.04)	0.011
Sleep trouble							
No	605/4,084	Reference		Reference		Reference	
Yes	301/1,753	1.26 (1.06–1.50)	0.008	1.13 (0.93–1.37)	0.214	1.04 (0.87–1.25)	0.651

HR, Hazard Ratio; CI, Confidence interval.

Model 1: Adjusted for age, sex, and race.

Model 2: Adjusted for age, sex, race, body Mass Index, uric acid, insurance, education, smoking status, alcohol consumption status, poverty-income ratio and physical activity.

Model 3:Adjusted for age, sex, race, body Mass Index, uric acid, insurance, education, smoking status, alcohol consumption status, poverty-income ratio, physical activity, cancer, gout, diabetes mellitus, hypertension, chronic kidney disease and cardiovascular disease.

### 3.3 Nonlinear association between sleep duration and all-cause mortality in hyperuricemic individuals

**Figure 2:**
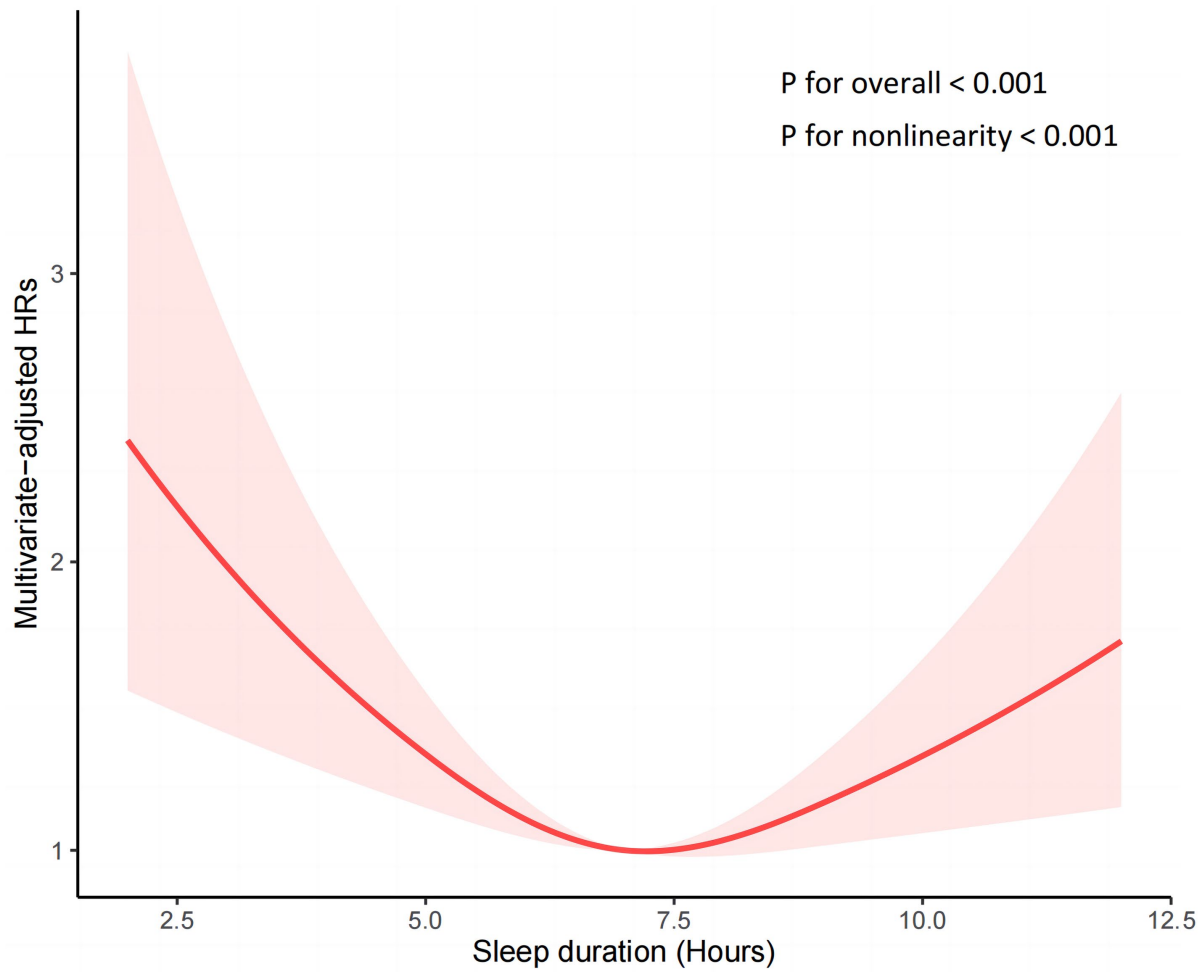
Nonlinear relationship between sleep duration and all-cause mortality by restricted cubic spline fitting. Adjusted for age, sex, race, body mass index, uric acid, insurance, education, smoking status, alcohol consumption status, poverty-income ratio, physical activity, cancer, gout, diabetes mellitus, hypertension, chronic kidney disease and cardiovascular disease.

To further examine the nonlinear association, we used RCS analysis in a fully adjusted Cox proportional hazard model, which demonstrated a U-shaped association (nonlinear *p* < 0.001) between sleep duration and the all-cause mortality risk ratio, as shown in Figure 2. The inflection point for the association between sleep duration and the HR of all-cause mortality is 7.23 h. To further investigate the trends and strength of the association between sleep duration and the HR of all-cause mortality on either side of the inflection point, we used a segmented model for analysis. The findings from the likelihood ratio test indicate that the segmented model fits the data better compared to the overall model. When sleep duration was less than 7.23 h, sleep duration was negatively correlated with the risk ratio of all-cause death, and the risk of all-cause death was decreased by 12.1% for each hour increase in sleep duration (HR:0.879, 95%CI: 0.788, 0.981, *p* = 0.022). When sleep duration was greater than 7.23 h, the risk of all-cause death increased by 18.7% for each additional hour of sleep duration (HR:1.187, 95%CI: 1.066, 1.320, *p* = 0.002), as shown in Table 3.

**Table 3:** Threshold effect analysis of sleep duration on all-cause mortality in participants with hyperuricemia.

	Adjusted HR (95%CI)	*p*-value
All-cause mortality		
Total	0.975 (0.937–1.014)	0.207
Segmented cox proportional hazards model		
Inflection point	7.23	
sleep duration<7.23	0.879 (0.788–0.981)	0.022
sleep duration≥7.23	1.187 (1.066–1.320)	0.002
P for Log-likelihood ratio	<0.001	

HR, Hazard Ratio; CI, Confidence interval.

The model was adjusted for age, sex, race, body mass index, uric acid, insurance, education, smoking status, alcohol consumption status, poverty-income ratio, physical activity, cancer, gout, diabetes mellitus, hypertension, chronic kidney disease and cardiovascular disease.

### 3.4 Subgroup and sensitivity analyses

The subgroup analyses examining the impact of age, sex, and race on the study results can be found in Supplementary Tables 6, 7. The analysis results indicate a notable interaction between sleep duration and race (P for interaction = 0.044). Specifically, when compared to those with normal sleep duration, the impact of insufficient sleep (HR:1.432; 95% CI: 1.148, 1.786; *p* = 0.001) and excessive sleep (HR:1.599; 95% CI: 1.095, 2.336; *p* = 0.015) on all-cause mortality is more pronounced among non-Hispanic White individuals. In the subgroup analyses of sleep duration by age and gender, no significant interactions were detected, nor were any significant interactions found regarding sleep trouble with age, gender, and race (all P for interaction >0.05), indicating that the results remain consistent across these different subgroups. To maintain the robustness of the research findings, we further excluded cases with missing covariates and hyperuricemia individuals with cancer in the sensitivity analysis, and obtained results similar to those before. The specific data and results can be found in Supplementary Tables 8, 9.

## 4 Discussion

This study uncovers the link between sleep duration and all-cause mortality in hyperuricemic individuals for the first time. The results of the study showed that all-cause mortality rose in hyperuricemic individuals with shorter and longer sleep duration compared with hyperuricemic individuals with normal sleep duration, and the two showed a U-shaped association. The inflection point for the relationship between sleep duration and all-cause mortality was 7.23 h. Our findings emphasize that normal sleep duration is an important prognostic factor for reducing all-cause mortality in hyperuricemic individuals. An intriguing point is that our findings were not what we expected, all-cause mortality in hyperuricemic individuals was not reduced by a sustained increase in sleep duration, but rather was U-shapedly correlated with sleep duration. Although most earlier research indicates a negative relationship between sleep duration and serum UA levels, this study found no notable variation in serum UA levels across participants with different sleep durations in the baseline data. This suggests that the U-shaped relationship between sleep duration and all-cause mortality in hyperuricemic individuals is not significantly affected by changes in serum UA levels, and that this association is more likely to result from the combined effect of all-cause mortality in the body due to a common or related pathophysiologic basis between higher UA levels and unhealthy sleep conditions.

Hyperuricemia and unhealthy sleep conditions are linked to the onset and development of a variety of diseases, including CVD, DM, metabolic syndrome, neurodegenerative diseases and CKD (1, 3, 26–28). The impact of hyperuricemia and unhealthy sleep conditions on disease is multifaceted, with both risk factors leading to common pathophysiological changes, including a pro-inflammatory response, increased oxidative stress and metabolic disturbances. Studies have shown that extracellular UA acts as an antioxidant *in vivo*, whereas higher levels of UA are transformed into an oxidative stressor, increasing oxidative stress and causing a variety of pathophysiological responses, including the promotion of inflammatory factor production, induction of inflammatory responses, as well as DNA damage, oxidization, and cell apoptosis (29). Both insufficient and excessive sleep promote inflammatory responses in the body, leading to chronic systemic low-grade inflammation, which further induces oxidative stress and reduces cellular antioxidant capacity (30, 31). Hyperuricemia is linked to metabolic syndrome and heightens the risk of its development. In addition, hyperuricemia leads to disorders of lipid metabolism, especially hypertriglyceridemia, and further promotes the accumulation of lipids in the body (32). Similarly, sleep deprivation causes dyslipidemia and increased insulin resistance in the body (33). These same pathophysiologic underpinnings promote the onset and development of diseases linked to unhealthy sleep conditions in hyperuricemic individuals, which leads to higher all-cause mortality in hyperuricemic individuals.

We note that not only is there an effect of sleep duration itself on all-cause mortality in hyperuricemic individuals, but that circadian rhythm disruption associated with insufficient and excessive sleep can likewise exacerbate this risk. Studies have shown that UA, as a compound, has a significant circadian rhythm in the human body (34), and that disrupted circadian rhythms are strongly associated with UA in the progression of various diseases, including CVD, cancer, metabolic syndrome, neurodegenerative diseases, and aging (35). Thus, for hyperuricemic individuals, a normal sleep duration not only improves the pathophysiologic response of the patient per se, but also effectively reduces the damage caused by circadian rhythm disturbances due to both insufficient and excessive sleep, thereby reducing the patient’s all-cause mortality.

Among the baseline characteristics of hyperuricemic individuals, individuals with long sleep duration were more likely to have inactive physical activity levels, while individuals with short sleep duration were more likely to have extremely active physical activity levels. Studies have shown that maintaining appropriate physical activity is effective in reducing all-cause mortality, CVD mortality, and cancer mortality (36). Excessive physical activity, however, can reduce the health benefits of exercise and even increase the risk of associated diseases, including atrial fibrillation, abnormal myocardial remodeling, and coronary artery calcification (37, 38). Additionally, there is evidence that appropriate physical activity improves sleep quality, while high intensity physical activity has no significant effect on sleep quality (39). High-quality sleep has many health benefits, including reduced incidence of CVD, reduced muscle strength impairment, enhanced cognitive and memory performance, and improved mental health (40–42). Thus, compared to the relatively active level of physical activity and good sleep quality of hyperuricemic individuals with normal sleep duration, individuals with too short and too long sleep duration are more likely to have an increased risk of developing related diseases due to inactive or extremely active physical activity and poorer sleep quality, and to further increase all-cause mortality in hyperuricemic individuals. It is worth noting that mental health problems, which is closely related to sleep duration and quality, also has a significant impact on an individual’s all-cause mortality. In a study of the general population in Spain, it was shown that those who slept less than 6 h were at greater risk for mental health problems (43). In a longitudinal study from China, poorer sleep quality, sleep deprivation on weekdays, and longer catch-up sleep on weekends were found to be strongly associated with the emergence of anxiety and depressive symptoms (44). In the college student population, shorter sleep duration as well as poorer sleep quality were associated with more mental health problems (45). Evidence suggests that poorer mental health is associated with increased all-cause mortality and premature death (46), whereas a healthy mental state is associated with lower all-cause mortality (47). Therefore, for hyperuricemic individuals, maintaining a normal sleep schedule has a positive effect on maintaining a healthy state of mental health, thereby reducing all-cause mortality in hyperuricemic individuals.

Our study determined that the optimal sleep duration for hyperuricemic individuals is 7.23 h, and hyperuricemic individuals at this sleep duration have the lowest all-cause mortality rate, making this inflection point clinically significant. Maintaining an optimal sleep duration in hyperuricemic individuals helps to reduce the body’s inflammatory response, oxidative stress, and metabolic disorders, and inhibit the onset and progression of related diseases, thereby achieving a reduction in the patient’s all-cause mortality. The optimal sleep duration identified in this study can guide the management of sleep duration in hyperuricemic individuals in clinical practice. Healthcare professionals ought to be mindful of the sleep duration of hyperuricemic individuals and make recommendations or reasonable interventions for individuals’ sleep duration. In addition, in clinical practice, individualized sleep management programs need to be developed according to the specific conditions of individuals to actively improve the prognosis of survival in hyperuricemic individuals.

Not only does this study provide important clinical insights, it also has several noteworthy strengths. First, this study was based on a nationally representative sample of U.S. adults for further analysis, which made our sample size sufficiently large and representative. In addition, our study controlled for some potential confounders, which made our findings more reliable, and the results of the sensitivity analyses indicated that the relationship between all-cause mortality and sleep duration in hyperuricemic individuals stayed relatively constant. Nevertheless, this study has some limitations. The UA level in this study was measured only once, and UA level is a dynamic variable that is highly influenced by diet, medication, and lifestyle habits; therefore, the UA data in this study do not provide a comprehensive picture of an individual’s UA level. The sleep duration used in this study was obtained by self-report, which may lead to some subjective bias, and our study did not consider the effect of sleep quality on all-cause mortality. Objective sleep measurement tools should be applied in future studies to comprehensively assess the effects of sleep duration and sleep quality on all-cause mortality. In this study, various sleep problems were uniformly classified as “trouble sleeping” for analysis. This may have masked the true relationships between different specific types of trouble sleeping, hyperuricemia, and all - cause mortality. Due to data limitations, we were unable to conduct a detailed sub - analysis of these differences. As a result, when interpreting the findings, we might not accurately capture the complex connections among them. This could make the potentially existing associations ambiguous and even lead to the conclusion of no correlation. In addition, it was not possible to include all confounders in our study due to data availability limitations.

## 5 Conclusion

The findings of this research suggest that sleep duration is strongly associated with all-cause mortality in hyperuricemic individuals. Among our nationally representative cohort of hyperuricemic individuals, we observed a U-shaped association between sleep duration and all-cause mortality in hyperuricemic individuals. All-cause mortality was lowest when sleep duration was 7.23 h, decreased by 12.1% for each additional hour of sleep when sleep duration was less than 7.23 h, and increased by 18.7% for each additional hour of sleep when sleep duration exceeded 7.23 h. However self-reported sleep trouble was not associated with all-cause mortality. Therefore, the focus should be on sleep duration in hyperuricemic individuals to actively improve their prognostic survival.

## Data Availability

The datasets presented in this study can be found in online repositories. The names of the repository/repositories and accession number(s) can be found at: https://www.cdc.gov/nchs/nhanes/index.htm.
