# Supplementary material for: Association between sleep duration, sleep trouble and all-cause mortality in individuals with hyperuricemia in the United States
Source: Front Public Health. 2025 Mar 26;13:1521372. doi: 10.3389/fpubh.2025.1521372 (PMC11979105; doi:10.3389/fpubh.2025.1521372)
Supplement: Supplementary file 1 [file Table_1.docx]

Supplementary Material

**Association between sleep duration, sleep trouble and all-cause mortality in individuals with hyperuricemia in the United States**

**Supplementary Table 1** Specific questions related to recording sleep time, along with their corresponding years and variable names

**Supplementary Table 2** Specific questions related to recording trouble sleeping, along with their corresponding years and variable names

**Supplementary Table 3** Criteria for categorizing levels of physical activity

**Supplementary Table 4** The definitions of hypertension, DM, and CKD

**Supplementary Table 5** The numbers and percentages of missing covariate data

**Supplementary Table 6** Subgroup analysis for the association between sleep time with all-cause mortality in patients with hyperuricemia

**Supplementary Table 7** Subgroup analysis for the association between trouble sleeping with all-cause mortality in patients with hyperuricemia

**Supplementary Table 8** Cox proportional hazards model for the association between sleep time, trouble sleeping, and all-cause mortality in patients with hyperuricemia (exclude participants with missing covariates)

**Supplementary Table 9** Cox proportional hazards model for the association between sleep time, trouble sleeping, and all-cause mortality in patients with hyperuricemia (exclude participants with cancer)

**Supplementary Table 1** Specific questions related to recording sleep time, along with their corresponding years and variable names.

| **Year** | **Variable Name** | **Questions** |
| --- | --- | --- |
| 2007-2008 | SLD010H | How much sleep {do you/does SP} usually get at night on weekdays or workdays? |
| 2009-2010 | SLD010H | How much sleep {do you/does SP} usually get at night on weekdays or workdays? |
| 2011-2012 | SLD010H | How much sleep {do you/does SP} usually get at night on weekdays or workdays? |
| 2013-2014 | SLD010H | How much sleep {do you/does SP} usually get at night on weekdays or workdays? |
| 2015-2016 | SLD012 | How much sleep {do you/does SP} usually get at night on weekdays or workdays? |
| 2017-2018 | SLD012 & SLD013 | Number of hours usually sleep on weekdays or workdays.  Number of hours usually sleep on weekends or non-workdays. |

**Supplementary Table 2** Specific questions related to recording trouble sleeping, along withtheir corresponding years and variable names.

| **Year** | **Variable Name** | **Questions** |
| --- | --- | --- |
| 2007-2008 | SLQ060 | {Have you/Has SP} ever told a doctor or other health professional that {you have/s/he has} trouble sleeping? |
| 2009-2010 | SLQ060 | {Have you/Has SP} ever told a doctor or other health professional that {you have/s/he has} trouble sleeping? |
| 2011-2012 | SLQ060 | {Have you/Has SP} ever told a doctor or other health professional that {you have/s/he has} trouble sleeping? |
| 2013-2014 | SLQ060 | {Have you/Has SP} ever told a doctor or other health professional that {you have/s/he has} trouble sleeping? |
| 2015-2016 | SLQ050 | {Have you/Has SP} ever told a doctor or other health professional that {you have/s/he has} trouble sleeping? |
| 2017-2018 | SLQ050 | {Have you/Has SP} ever told a doctor or other health professional that {you have/s/he has} trouble sleeping? |

**Supplementary Table 3** Criteria for categorizing levels of physical activity

| **Classification** | **Classification criteria** |
| --- | --- |
| Inactive | 0 MET-min/week |
| Low-active | >0, ≤600 MET-min/week |
| Highly active | >600, ≤1500 MET-min/week |
| Extremely highly active | >1500 MET-min/week |

Abbreviation: MET, Metabolic equivalent of task.

**Supplementary Table 4** The definitions of hypertension, DM, and CKD

| **Disease** | **Diagnostic basis** |
| --- | --- |
| DM | A diagnosis by a physician or other health professional |
|  | Random blood sugar ≥11.1 (mmol/L) |
|  | Glycosylated hemoglobin (%) greater than 6.5 |
|  | Use of diabetes medications or insulin |
| Hypertension | A diagnosis by a doctor or other health professional |
|  | An average blood pressure ≥130/80 mmHg |
|  | Use of hypertension medications |
| CKD | Glomerular filtration rate <60 mL/min/1.73 m^2^ |
|  | Urinary albumin to creatinine ratio >30 mg/g |

Abbreviation: DM, Diabetes mellitus; CKD, Chronic kidney disease.

**Supplementary Table 5** The numbers and percentages of missing covariate data

| **Covariate** | **Numbers** | **Percentages (%)** |
| --- | --- | --- |
| Alcohol consumption status | 639 | 10.95 |
| Poverty-income ratio | 569 | 9.75 |
| Body Mass Index | 104 | 1.78 |
| Insurance | 13 | 0.22 |
| Gout | 9 | 0.15 |
| Chronic kidney disease | 7 | 0.98 |
| Education | 6 | 0.10 |
| Cancer | 5 | 0.09 |
| Diabetes mellitus | 5 | 0.09 |
| Somke | 3 | 0.05 |
| Cardiovascular disease | 2 | 0.03 |

**Supplementary Table 6** Subgroup analysis for the association between sleep time with all-cause mortality in patients with hyperuricemia

|  | **Sleep time** | | | | | | | | |
| --- | --- | --- | --- | --- | --- | --- | --- | --- | --- |
|  | **7-9** | | | **<7** | | **>9** | | P for interaction | |
|  |  | |  | HR(95%CI) | p value | HR(95%CI) | p value |  |  |
| **Age** |  |  | |  |  |  |  | 0.474 |  |
| 20-60 | Reference | | | 1.321 (0.812, 2.148) | 0.262 | 1.860 (0.823,4.203) | 0.136 |  |  |
| >60 | Reference | | | 1.212 (0.986,1.489) | 0.068 | 1.462 (1.015,2.107) | 0.041 |  |  |
| **Sex** |  |  | |  |  |  |  | 0.540 |  |
| Male | Reference | | | 1.068 (0.810,1.410) | 0.640 | 1.445 (0.936,2.231) | 0.097 |  |  |
| Female | Reference | | | 1.436 (1.090,1.893) | 0.010 | 1.497 (0.921,2.433) | 0.104 |  |  |
| **Race** |  |  | |  |  |  |  | 0.044 |  |
| Non-Hispanic white | Reference | | | 1.432 (1.148,1.786) | 0.001 | 1.599 (1.095,2.336) | 0.015 |  |  |
| Non-Hispanic black | Reference | | | 0.973 (0.752,1.258) | 0.834 | 1.156 (0.668,2.001) | 0.605 |  |  |
| Mexican American | Reference | | | 0.910 (0.428,1.935) | 0.807 | 0.788 (0.341,1.819) | 0.577 |  |  |
| Other | Reference | | | 0.920 (0.569,1.487) | 0.733 | 1.511 (0.588,3.886) | 0.391 |  |  |

Abbreviation: HR, Hazard Ratio; CI, Confidence Interval.

Adjusted for age, sex, race, bmi, uric acid, insurance, education, smoking status, alcohol consumption status, poverty-income ratio, physical activity, cancer, gout, diabetes mellitus, hypertension, chronic kidney disease and cardiovascular disease.

**Supplementary Table 7** Subgroup analysis for the association between trouble sleeping with all-cause mortality in patients with hyperuricemia

|  | **Trouble sleeping** | | | | |  |
| --- | --- | --- | --- | --- | --- | --- |
|  | **NO** | | | **YES** | | P for interaction |
|  |  | |  | HR(95%CI) | P value |  |
| **Age** |  |  | |  |  | 0.093 |
| 20-60 | Reference | | | 1.202 (0.781,1.849) | 0.404 |  |
| >60 | Reference | | | 0.956 (0.776,1.179) | 0.675 |  |
| **Sex** |  |  | |  |  | 0.993 |
| Male | Reference | | | 0.983 (0.770,1.254) | 0.889 |  |
| Female | Reference | | | 1.025 (0.783,1.343) | 0.857 |  |
| **Race** |  |  | |  |  | 0.776 |
| Non-Hispanic white | Reference | | | 1.043 (0.843,1.292) | 0.697 |  |
| Non-Hispanic black | Reference | | | 0.902 (0.652,1.250) | 0.537 |  |
| Mexican American | Reference | | | 1.034 (0.489,2.186) | 0.931 |  |
| Other | Reference | | | 1.402 (0.886,2.219) | 0.150 |  |

Abbreviation: HR, Hazard Ratio; CI, Confidence Interval.

Adjusted for age, sex, race, bmi, uric acid, insurance, education, smoking status, alcohol consumption status, poverty-income ratio, physical activity, cancer, gout, diabetes mellitus, hypertension, chronic kidney disease and cardiovascular disease.

**Supplementary Table 8** Cox proportional hazards model for the association between sleep time, trouble sleeping, and all-cause mortality in patients with hyperuricemia (exclude participants with missing covariates)

|  | **Fully adjusted model** | |
| --- | --- | --- |
|  | HR (95%CI) | P value |
| **Sleep time** |  |  |
| Normal (7-9 hours) | Reference | |
| Short (< 7 hours) | 1.22 (0.97,1.53) | 0.087 |
| Long (>9 hours) | 1.52 (1.04,2.22) | 0.030 |
| **Trouble sleeping** |  |  |
| No | Reference | |
| Yes | 1.03 (0.84,1.27) | 0.758 |

Abbreviation: HR, Hazard Ratio; CI, Confidence Interval.

Adjusted for age, sex, race, bmi, uric acid, insurance, education, smoking status, alcohol consumption status, poverty-income ratio, physical activity, cancer, gout, diabetes mellitus, hypertension, chronic kidney disease and cardiovascular disease.

**Supplementary Table 9** Cox proportional hazards model for the association between sleep time, trouble sleeping, and all-cause mortality in patients with hyperuricemia (exclude participants with cancer)

|  | **Fully adjusted model** | |
| --- | --- | --- |
|  | HR (95%CI) | P value |
| **Sleep time** |  |  |
| Normal (7-9 hours) | Reference | |
| Short (< 7 hours) | 1.35 (1.03,1.78) | 0.032 |
| Long (>9 hours) | 1.62 (1.12,2.34) | 0.010 |
| **Trouble sleeping** |  |  |
| No | Reference | |
| Yes | 1.09 (0.87,1.37) | 0.438 |

Abbreviation: HR, Hazard Ratio; CI, Confidence Interval.

Adjusted for age, sex, race, bmi, uric acid, insurance, education, smoking status, alcohol consumption status, poverty-income ratio, physical activity, cancer, gout, diabetes mellitus, hypertension, chronic kidney disease and cardiovascular disease.
